# Detection of Alpha- and Betacoronaviruses in Frugivorous and Insectivorous Bats in Nigeria

**DOI:** 10.3390/pathogens11091017

**Published:** 2022-09-07

**Authors:** Uwem George, Oluwadamilola George, Arthur Oragwa, Babatunde Motayo, Joshua Kamani, Andrew Adamu, Oluyomi Sowemimo, Richard Adeleke, Samson Abalaka, Nuhu Sani, Judith Oguzie, Philomena Eromon, Onikepe Folarin, Anise Happi, Isaac Komolafe, Christian Happi

**Affiliations:** 1 African Centre of Excellence for Genomics of Infectious Diseases, Redeemer’s University, Ede 232102, Osun State, Nigeria; 2 Department of Biological Sciences, Faculty of Natural Sciences, Redeemer’s University, Ede 232102, Osun State, Nigeria; 3 Ibadan Diagnostic and Epidemiology Laboratory, National Veterinary Research Institute, Mokola, Ibadan 200212, Oyo State, Nigeria; 4 Department of Veterinary Microbiology, Faculty of Veterinary Medicine, University of Jos, Jos 930003, Plateau State, Nigeria; 5 Department of Medical Microbiology, Federal Medical Centre, Abeokuta 110222, Ogun State, Nigeria; 6 Parasitology Division, National Veterinary Research Institute (NVRI), PMB 01, Vom 930103, Plateau State, Nigeria; 7 Australian Institute of Tropical Health and Medicine, Division of Tropical Health and Medicine, James Cook University, Townsville, QLD 4811, Australia; 8 College of Public Health, Medical and Veterinary Sciences, James Cook University, 1 James Cook Drive, Bebegu Yumba Campus, Douglas, QLD 4811, Australia; 9 Department of Veterinary Public Health and Preventive Medicine, University of Abuja, Abuja 900105, Federal Capital Territory, Nigeria; 10 Department of Zoology, Faculty of Science, Obafemi Awolowo University, Ile Ife 220005, Osun State, Nigeria; 11 Immunology and Infectious Diseases, College of Veterinary Medicine, Cornell University, New York, NY 14853, USA; 12 Department of Veterinary Microbiology, Faculty of Veterinary Medicine, University of Ibadan, Ibadan 200132, Oyo State, Nigeria; 13 Department of Veterinary Pathology, Faculty of Veterinary Medicine, University of Abuja, Abuja 900105, Federal Capital Territory, Nigeria

**Keywords:** coronavirus diversity, bats, zoonosis, surveillance, Nigeria

## Abstract

The rise of bat-associated zoonotic viruses necessitates a close monitoring of their natural hosts. Since the detection of severe acute respiratory syndrome coronavirus (SARS-CoV), it is evident that bats are vital reservoirs of coronaviruses (CoVs). In this study, we investigated the presence of CoVs in multiple bat species in Nigeria to identify viruses in bats at high-risk human contact interfaces. Four hundred and nine bats comprising four bat species close to human habitats were individually sampled from five states in Nigeria between 2019 and 2021. Coronavirus detection was done using broadly reactive consensus PCR primers targeting the RNA-dependent RNA polymerase (RdRp) gene of CoVs. Coronavirus RNA was detected in 39 samples (9.5%, CI 95%: [7.0, 12.8]), of which 29 were successfully sequenced. The identified CoVs in Nigerian bats were from the unclassified African alphacoronavirus lineage and betacoronavirus lineage D (*Nobecovirus*), with one sample from *Hipposideros ruber* coinfected with alphacoronavirus and betacoronavirus. Different bat species roosting in similar or other places had CoVs from the same genetic lineage. The phylogenetic and evolutionary dynamics data indicated a high CoV diversity in Nigeria, while host switching may have contributed to CoV evolution. Robust sentinel surveillance is recommended to enhance our knowledge of emerging and re-emerging coronaviruses.

## 1. Introduction

Bat (order Chiroptera) are mammals with over 1300 species across 20 families and 175 genera, accounting for over 20% of known mammalian species globally [1]. They are widely spread in nature and play a significant role in the biological diversity of various ecosystems [2]. Multiple studies have documented the role of bats as reservoirs of different viral agents of public health importance, including the progenitors of SARS-CoV and SARS-CoV-2, the causative agent for the COVID-19 pandemic [3,4,5]. The origin of SARS-CoV-2 and the possible role of intermediate animal host(s) in early animal-to-human transmission are unanswered questions associated with the COVID-19 pandemic. Several studies have revealed various CoVs in African bats [6,7,8,9]. However, there is a paucity of data on bat CoVs in Nigeria, where we only have information on betacoronavirus infection in bats from North-Central and South-West Nigeria [10,11].

Bats are hunted and eaten in some parts of Africa, including Nigeria [12,13,14,15,16]. Hunters in Ghana (Afram plains and Volta regions) have confessed to consuming *Eidolon helvum* bats with the decision to hunt bats based on their family tradition, further enhanced by economic necessity [15]. At the same time, *Rousettus aegyptiacus* is heavily hunted in eastern Nigeria, and several hunters in South-South Nigeria (Niger Delta region) were reported to hunt bats occasionally [16]. In southwestern Nigeria, the straw-coloured fruit bat is popular meat [12]. This close interaction between humans and bats may allow the large-scale emergence of novel virus types and species with unpredictable pathogenicity and clinical impacts. Thus, proactive measures, including surveillance and enhanced pathogen discovery techniques in emerging infectious disease “hotspots”, especially when there are no known epidemics, might improve the early recognition of potential outbreaks and the detection of novel pathogens. This research aimed to catalogue coronavirus diversity in Nigerian bats, a critical component for public health measures to prevent future outbreaks that other bat coronaviruses may cause.

## 2. Materials and Methods

### 2.1. Study Area and Sample Collection

**Figure 1:**
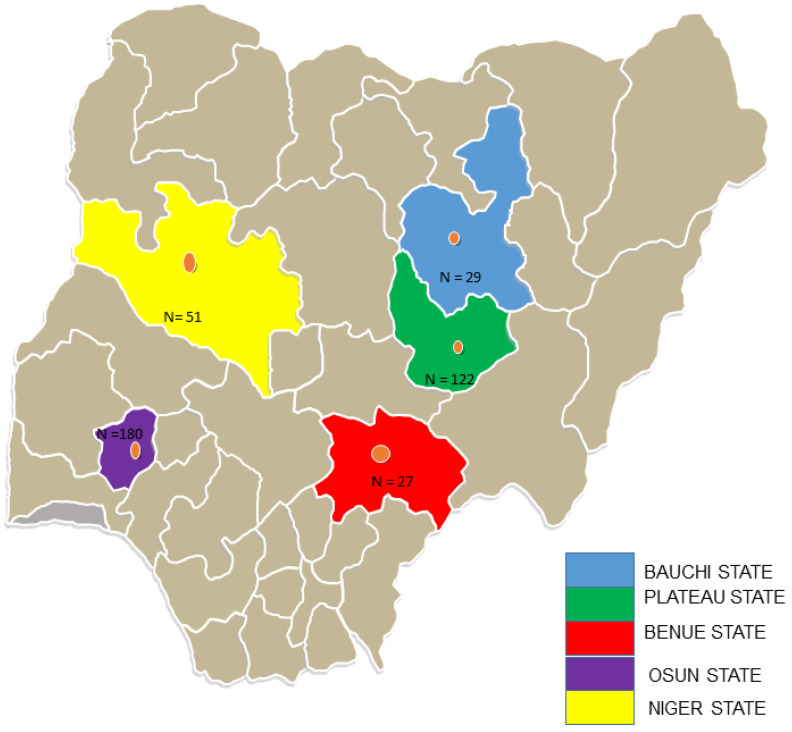
Map of Nigeria showing the five states where bat samples were collected.

Samples were collected from insectivorous and free-ranging fruit bats in five states in Nigeria between November 2019 and May 2021 (Figure 1). Bats were trapped around fruit trees and human dwellings using harp traps and mist nets. Each captured bat was assessed, and morphological characteristics such as weight (g), forearm and tibia length (mm), sex, reproductive state, and age were recorded to determine bat species. Oral and rectal swabs were collected and placed into tubes containing 1 mL of virus transport medium. A few of the trapped bats were humanely euthanised under a veterinarian’s supervision in full compliance with the local ethical and legal guidelines, and voucher specimens were collected. All experiments were conducted in a microbiological safety station with personal protective equipment, a mask, and a visor. All samples were immediately transferred to −20 °C containers before being transported to the laboratory at the African Centre of Excellence for Genomics of Infectious Diseases (ACEGID), Redeemer’s University, Nigeria and stored at −80 °C until processed.

### 2.2. Nucleic Acid Extraction

Total RNA was extracted from oral and rectal swabs using the QIAamp^®^ Viral RNA extraction kit (Qiagen^®^, Hilden, Germany) according to the manufacturer’s instructions with an elution volume of 60 μL. According to the manufacturer’s manual, DNA was isolated from faecal swabs using the DNeasy Blood and Tissue Kit (#69506; Qiagen).

### 2.3. Molecular Confirmation of Bat Species

Bat host species identification was confirmed for each bat in which coronavirus was detected by selectively amplifying segments of vertebrate mitochondrial cytochrome oxidase subunit 1 (COI) and cytochrome b (Cyt b) mtDNA [17]. Briefly, a fragment of approximately 700 bp of COI (primer pair COI_long-f 5′-AACCACAAAGACATTGGCAC-3′ and COI_long-r 5′-AAGAATCAGAATARGTGTTG-3′) and 520 bp of Cytb (primer pair Cytb-f 5′-GAGGMCAAATATCATTCTGAGG-3′ and Cytb-r 5′-TAGGGCVAGGACTCCTCCTAGT-3′) was amplified. PCR products were purified using the QIAquick^®^ Gel and PCR Clean-up kit and sequenced directly using an automated ABI 3500xl DNA Sequencer at ACEGID, Redeemer’s University, Ede, Nigeria. Nucleotide sequences were edited using BioEdit Sequence Alignment Editor Version 7.2.6, and a BLASTn search was done to identify bat species. For samples with low similarity (<90%) hits with sequences in GenBank, an alignment with reference sequences was done using the MUSCLE program in MEGA 11 software with default settings [18,19], and phylogenetic trees were constructed using the maximum likelihood method.

### 2.4. RT-PCR Screening for Detection of Coronavirus RNA using Heminested Reverse-Transcription PCR (RT-PCR) and Sanger Sequencing

The detection of bat coronaviruses was done using heminested reverse-transcription PCR (RT-PCR) with broadly reactive consensus PCR primers targeting the RNA-dependent RNA polymerase (RdRp) gene of different CoVs as previously described [10]. The synthesis of cDNA was carried out using Superscript IV First-Strand Synthesis kit (Invitrogen) followed by the nested PCR. The amplified product of 328 bp was visualised using 2% agarose gel electrophoresis. The RdRp PCR products were purified using the QIAquickn^®^ Gel and PCR Clean-up kit and sequenced directly using an automated ABI 3500xl DNA Sequencer available at ACEGID.

### 2.5. Phylogenetic Analysis

MAFFT online service [20] was used to align the sequences, and MEGA version 11 [19] was used to build a phylogenetic tree using the maximum likelihood with the gamma-distributed Hasegawa–Kishino–Yano (HKY+G) model [21], with 1000 bootstrap replications to assess phylogenetic robustness. The tree was visualised using Interactive Tree of Life (iTOL) v5 [22]. We aligned every unique pair of sequences, estimated sequence pairwise identity between sequences from our work, and published references using the Sequence Demarcation Tool (SDT) [23].

Phylogenetic trees were also generated by IQ-TREE version 1.6.12 5 [24] using a partition model [25] with ModelFinder [26] and ultrafast bootstrap (1000 replicates) [27] to explore the reconstruction of ancestral-state phylogeographic transmission across countries and host species. Coding genes were partitioned into first + second and third codon positions. Results were visualised using Microreact (https://microreact.org/ (accessed on 1 March 2022)) [28].

### 2.6. Statistical Analysis

The Wilson method [29] in the Epitools calculator (http://epitools.ausvet.com.au (accessed on 1 March 2022)) was used to calculate confidence intervals for prevalence.

## 3. Results

### 3.1. Bats Samples Collected and Prevalence of Coronavirus

From 2019 to 2021, 409 bat samples were collected from six roosting sites in five states in Nigeria (Table 1). After morphological inspection and sequence analysis of the *Cyt b* and COI mtDNA, these bats were classified into four species: *E. helvum*, *Hipposideros ruber*, *Mops condylurus*, and *Chaerephon* sp.

Using nested RT-PCR, coronaviruses were detected in 39 samples, giving an overall detection rate of 9.5% (CI 95%: 7.0–12.8). The 39 samples identified as CoV positive included 8 (6.6%) among the 122 *E. helvum* from the Jos roosting site, 3 (3.5%) among the 86 *H. ruber* from the Ife roosting site, 3 (3.2%) among the 93 *E. helvum* from the Ife roosting site, 6 (22.2%) among the 27 *M. condylurus* from the Gboko/Benue roosting site, and 19 (37.3%) among the 51 *Chaerephon* sp. from the Paiko/Niger roosting site (Table 1). Positive samples were detected in bats from all the roosting sites except *E. helvum* samples collected in 2019 and 2020 from Bauchi and Ede. Bats positive for CoVs were captured in May and June 2020, October–December 2020 and January–March 2021. Coronaviruses were detected in both male and female bats irrespective of age. Coronaviruses were also seen in the pooled rectal and oral swabs (in the case of samples with low volume) and unpooled rectal and oral swabs, respectively (Table 2).

### 3.2. Molecular Characterization of Identified CoVs and Estimation of Divergence Time

**Figure 2:**
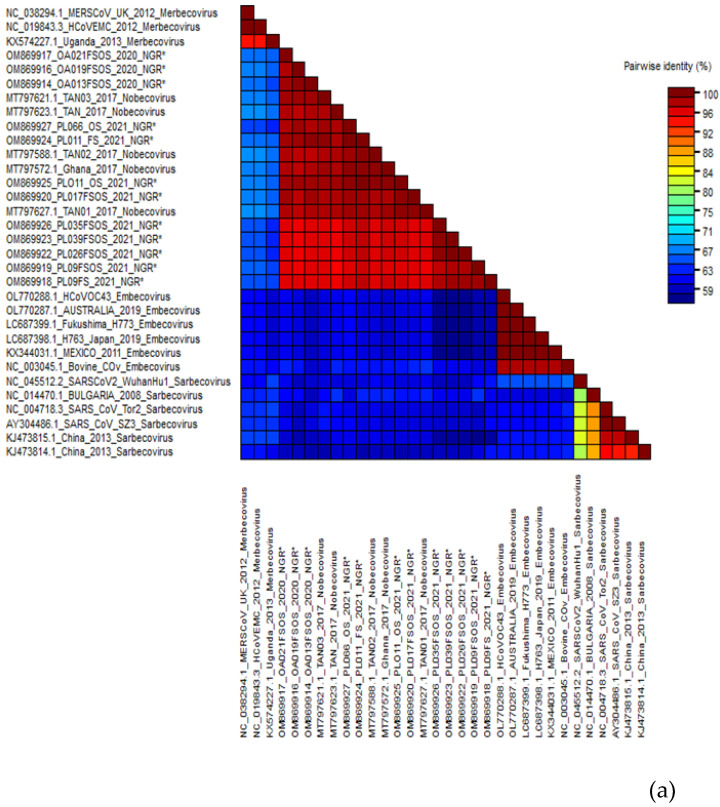

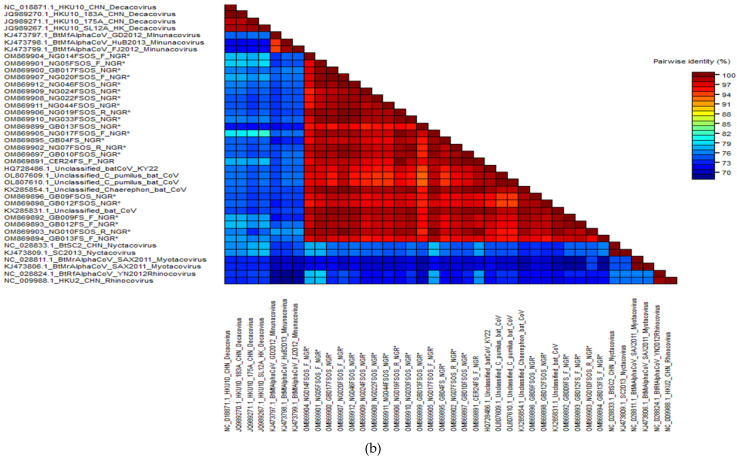
(**a**) Alignment result of β-CoV sequences and estimated sequence pairwise identity between sequences from this study and published references (representative members from each β-CoV subgenera and close match from BLASTn search) using the Sequence Demarcation Tool. New sequences derived from the current study are denoted with an asterisk. (**b**) Alignment result of α-CoV sequences and estimated sequence pairwise identity between sequences from this study and published references (representative members from each α-CoV subgenera and close match from BLASTn search) using the Sequence Demarcation Tool. New sequences derived from the current study are denoted with an asterisk.

Of the 39 RT-PCR-positive bat samples screened, 29 bat samples were successfully sequenced, and the nucleotide sequences were compared to those in the public database using the “Blastn” algorithm of NCBI BLAST and SDT. Of the 29 newly identified CoVs, 18 belonged to alphacoronaviruses (α-CoV) and 10 to betacoronaviruses (β-CoV). One sample from *H. ruber* bat (CER024_NGR) was coinfected with alphacoronavirus and betacoronavirus (Table 2). According to the findings, all the nucleotide sequences from β-CoV detected in this study were 91.7–98.7% identical to the *E. helvum* coronavirus in lineage D (*Nobecovirus*) previously reported in *E. helvum* bats in Cameroon, Ghana, Tanzania, and Kenya (Figure 2a). The α-CoV nucleotide sequences were 95.94–98.61% identical to the *Alphacoronavirus* genus (Chaerephon bat coronavirus) (Figure 2b).

**Figure 3:**
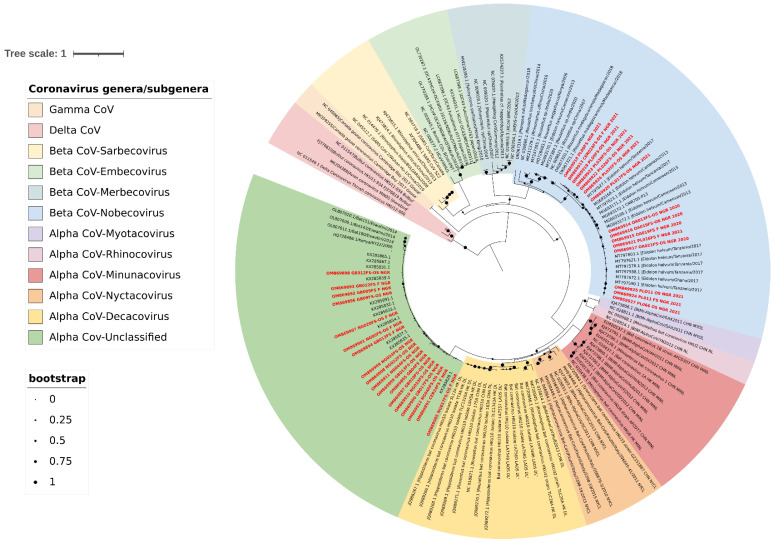
Maximum likelihood tree with gamma-distributed Hasegawa–Kishino–Yano (HKY+G) model based on 327 bp RdRp sequences, with 1000 bootstrap replications. Sequences reported in this study are in bold red font. The tree was visualised using Interactive Tree of Life (iTOL) v5 with midpoint rooting and each coronavirus lineage colour-coded as shown in the legend.

To determine the genetic relationships between the sequenced bat CoVs from this study and previously described CoVs, a phylogenetic analysis using the maximum likelihood technique was performed based on 327 bp RdRp truncated sequences. Phylogenetic analysis confirmed that all the β-CoV sequences from this study were in the Nobecovirus lineage, while the alphacoronavirus clustered within the unclassified African α-CoV lineage (Chaerephon bat coronavirus) (Figure 3).

**Figure 4:**
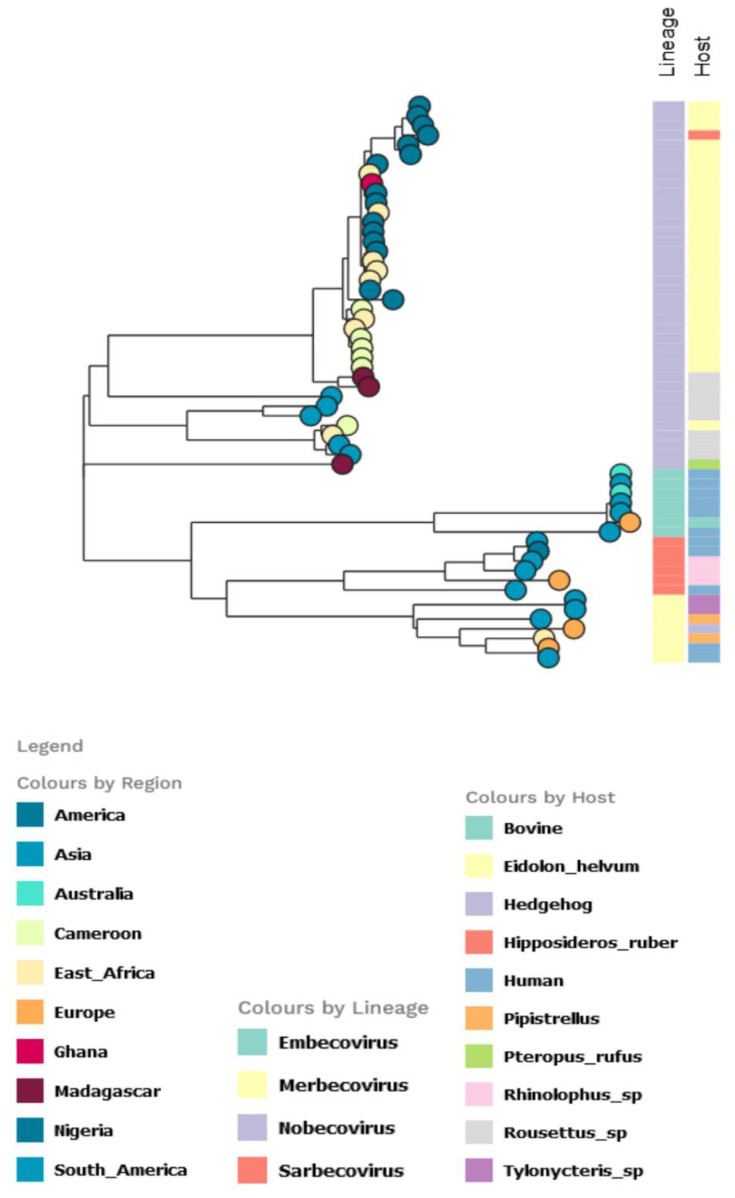
Partitioned model tree to explore phylogeographic transmission across countries and host species of partial RdRp gene region of β-CoV. The taxa are represented by their countries of origin with the lineage and host equally shown.

The partition model tree with host trait revealed that virtually all the *E. helvum* infected bats were infected with Nobecovirus, with the subgenera circulating mainly in *E. helvum, H. ruber*, and *Rousettu* sp. bats in Africa. In contrast, the other subgenera were equally spread in diverse bat species. All the Sarbecovirus isolates were from human hosts and *Rhinolophus* sp. except a single bovine isolate (Figure 4).

**Figure 5:**
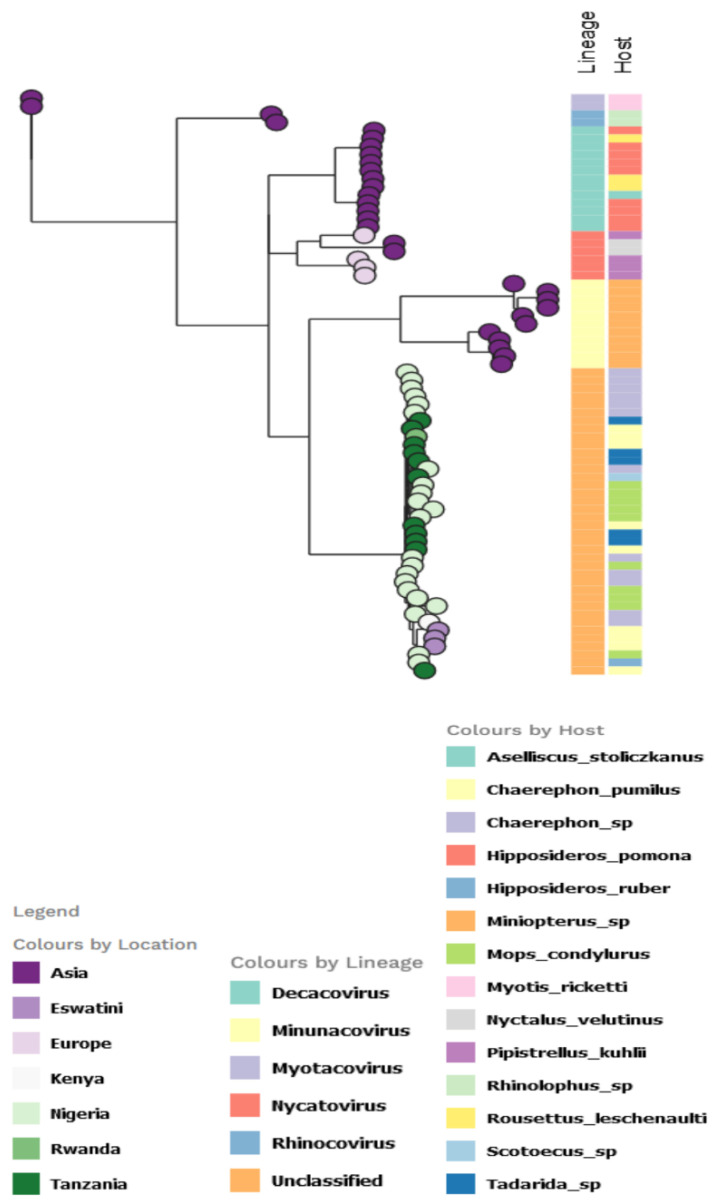
Partitioned model tree to explore phylogeographic transmission across countries and host species of partial RdRp gene region of α-CoV. The taxa are represented by their countries of origin with the lineage and host equally shown.

For the AlphaCoV partition tree, the unclassified African lineage showed a more diversified host species distribution, with the *Chaerephon* sp. having the highest distribution, followed by *M. condylurus* among the Nigerian bat viruses (Figure 5). The lineage also infected other bat species in Africa, including *Chaerephon pumilus* (in Eswatini and Kenya).

## 4. Discussion

In this study, we analysed samples from 409 bats collected from five states in Nigeria. We detected coronavirus RNA in 9.5% (39) specimens sampled from all bat species and observed a high CoV diversity in Nigerian bats. Different bat species roosting in similar or other places had CoVs from the same genetic lineage, suggesting that host switching may contribute to CoV evolution in Nigeria. All the betacoronaviruses belonged to the *E. helvum* coronavirus in lineage D (Nobecovirus). In contrast, the alphacoronavirus belonged to the unclassified Chaerephon bat coronavirus lineage, which is the first report of this virus in Nigerian bats.

The overall prevalence of CoV in bats of 9.5% (CI 95%: 7.0–12.8) in this study is consistent with reports of CoVs in bats from Ghana, Germany, and a recent global survey of coronaviruses in bats, rodents, and nonhuman primates [6,30,31]. However, in previous studies from Nigeria, infection rates were generally lower [10,11,32]. High rates were observed in two insectivorous bat species in the family *Molossidae* (6/27 *M. condylurus* and 19/51 *Chaerephon* sp.). However, it is probable that the high rates observed in these bat species may be related to the season in which the samples were obtained. Seasonal variations in infection prevalence are most likely influenced by density fluctuations during colony establishment or migration, affecting contact rates and disease dynamics. Furthermore, due to continuous interaction among individuals inside a maternity roost, virus transmission is more easily facilitated during the breeding season than at other times [30,33,34]. Additionally, as previously suggested [35,36,37], coronavirus transmission may be aided by the high colony density caused by the birth pulse. Subsequently, the seasonal surge of vulnerable juveniles could speed the spread of the virus throughout the colony, including the infection of adult bats. Thus, there is a need for data covering both breeding seasons and nonbreeding seasons to understand how coronaviruses are maintained in the Nigerian bat population.

Our study showed that Nigerian bats harboured phylogenetically structured CoVs, of both α-CoV and β-CoV subclades, clustering mostly by bat family. Host specificity has been widely reported for some bat CoVs subgenera; whereas β-D CoVs are mostly in *Pteropodidae,* β-C CoVs are associated mainly with *Vespertilionidae* [31,38]. Our findings of *Nobecovirus* (*E. helvum* bat coronavirus-like cluster) dominance among *Pteropodidae* are consistent with a prior study that found widespread *Nobecovirus* (Lineage D) circulation among fruit bats in some African countries [11,39]. The species-specific phylogenetic clustering observed among *E. helvum* bats suggests limited interspecies β-CoV transmission and host-specific evolution among these species of bats in Nigeria. However, detecting the virus in *H. ruber* in an abandoned basement around the *E. helvum* roosting site suggests the existence of potentially evolving virus strains, with a possible ability to cross the species barrier.

We observed a strong geographic influence on CoV diversity within the family *Molossidae*, which may have resulted from host switching. Host switching and coevolution have been reported as influential evolutionary mechanisms for African CoVs [7,8,31,40,41]. We found that genetically related CoVs were present in other bat species. For example, the Chaerephon bat coronavirus cluster was detected in *Chaerephon* sp. (Paiko, Niger state), *M. condylurus* (Gboko, Benue state) and *H. ruber* (CER/OAU-Ife/Osun state). Similar CoVs were seen in the same type of bat in different locations, as noted for *E. helvum-like* CoVs clusters detected in *E. helvum* from other sites, including Jos and Ife in Plateau and Osun states, respectively. These findings suggest that genetically diverse coronaviruses cocirculate among bats in various regions in Nigeria.

We observed an alpha- and betacoronavirus coinfection in a single *H. ruber* bat. Coinfections have previously been documented in African *R. aegyptiacus* bats, Hipposideros bat species and Asian insectivorous bats [8,42,43,44,45,46].

## 5. Conclusions

This study demonstrated high rates of CoVs in frugivorous and insectivorous bats in Nigeria, with significant genetic diversity. Phylogenetic and evolutionary dynamics data indicate a high CoV diversity in Nigeria, while host switching may contribute to CoV evolution. A robust sentinel surveillance is recommended to enhance our knowledge of emerging and re-emerging CoVs. Furthermore, future research should concentrate on locations and bat species where bat–human contact is expected or likely to become common due to climate change and other environmental factors.

## Figures and Tables

**Table 1:** Georeferenced location, percentage of samples positive for coronavirus RNA in various bat families, and species sampled in 2019–2021.

Location	Georeference	Year of Sample Collection	Family/Species	Coronavirus RNA (Prevalence, CI (Samples Collected))
Lim, Bauchi State	10°18′57” N, 9°50′39” E	2019	*Pteropodidae/E. helvum*	0.0–11.7 (29)
Ede, Osun State	7°45′36” N 4°26′50′’ E	2020	*Pteropodidae/E. helvum*	0.0–6.5 (2)
Gboko, Benue State	7°19′30” N 9°0′18′’ E	2020	*Molossidae/M. condylurus*	22.2. 10.6–40.8 (27)
OAU-Ife, Osun State	7°31′11′’ N 4°31′34′’ E	2020	*Pteropodidae/E. helvum*	3.3. 1.1–9.2 (92)
CER-OAU-Ife, Osun State	7°30′27′’ N 4°31′21′’ E	2020	*Hipposideridae/H. ruber*	0. 0–10.7 (32)
CER-OAU-Ife, Osun State	7°30′27′’ N 4°31′21′’ E	2021	*Hipposideridae/H. ruber*	5.6. 1.9–15.1 (54)
Jos Zoo/Museum, Plateau State	9°54′58.6” N 8°53′11.8′’ E	2021	*Pteropodidae/E. helvum*	6.6. 3.4–12.4 (122)
Paiko, Niger State	9°26′25.6” N 6°38′0.39” E	2021	*Molossidae/Chaerephon* sp.	37.3. 25.3–50.9 (51)

CI = confidence interval.

**Table 2:** Characteristics of bats infected with coronavirus.

Sample ID	Species	Sex	Age	Location	Collection Date	Type of Samples and CoV Detected			GenBank Accession Number
						Pooled			
						Rectal/Oral	Oral Swab	Rectal Swab	
CER024_NGR^1^	*H. ruber*	Male	Adult	OAU-IFE/OSUN Abandoned basement	2021	BetaCoV	-ve	AlphaCoV	OM869913/ OM869891
OA013_NGR	*E. helvum*	Female (lactating)	Adult	OAU-IFE/OSUN (On trees)	2020	BetaCoV	-ve	Positive (Sequence not exploitable)	OM869914
OA019_NGR	*E. helvum*	Female	Juvenile	OAU-IFE/OSUN (On trees)	2020	BetaCoV	-ve	BetaCoV	OM869915/ OM869916
OA021_NGR	*E. helvum*	Male	Adult	OAU-IFE/OSUN (On trees)	2020	BetaCoV	-ve	Positive (Sequence not exploitable)	OM869917
PL09_NGR	*E. helvum*	Male	Adult	JOS/PLATEAU (On trees)	2021	BetaCoV	-ve	BetaCoV	OM869918/ OM869919
PL017_NGR	*E. helvum*	Female	Adult	JOS/PLATEAU (On trees)	2021	BetaCoV	-ve	Positive (Sequence not exploitable)	OM869920
PL026_NGR	*E. helvum*	Male	Adult	JOS/PLATEAU (On trees)	2021	BetaCoV	-ve	BetaCoV	OM869921/ OM869922
PL039_NGR	*E. helvum*	Male	Adult	JOS/PLATEAU (On trees)	2021	BetaCoV	-ve	Positive (Sequence not exploitable)	OM869923
PL011_NGR	*E. helvum*	Female	Juvenile	JOS/PLATEAU (On trees)	2021	Positive (Sequence not exploitable)	BetaCoV	BetaCoV	OM869924/ OM869925
PL035_NGR	*E. helvum*	Male	Juvenile	JOS/PLATEAU (On trees)	2021	BetaCoV	-ve	Positive (Sequence not exploitable)	OM869926
PL066_NGR	*E. helvum*	Female	Adult	JOS/PLATEAU (On trees)	2021		BetaCoV		OM869927
GB009_NGR	*M. condylurus*	Female	Adult	The ceiling of a residential building	2020	AlphaCoV	-ve	AlphaCoV	OM869892/ OM869896
GB012_NGR	*M. condylurus*	Female	Juvenile	The ceiling of a residential building	2020	AlphaCoV	-ve	AlphaCoV	OM869893/ OM869898
GB013_NGR	*M. condylurus*	Female	Adult	The ceiling of a residential building	2020	AlphaCoV	-ve	AlphaCoV	OM869894/ OM869899
GB04_NGR	*M. condylurus*	Male	Juvenile	The ceiling of a residential building	2020	Positive (Sequence not exploitable)	-ve	AlphaCoV	OM869895
GB010_NGR	*M. condylurus*	Female	Adult	The ceiling of a residential building	2020	AlphaCoV	NT	NT	OM869897
GB017_NGR	*M. condylurus*	Female	Adult	The ceiling of a residential building	2020	AlphaCoV	NT	NT	OM869900
NG05_NGR	*Chaerephon* sp.	Male	Adult	The ceiling of a PHC facility	2021	AlphaCoV	NT	NT	OM869901
NG07_NGR	*Chaerephon* sp.	Female	Adult	The ceiling of a PHC facility	2021	AlphaCoV	NT	NT	OM869902
NG010_NGR	*Chaerephon* sp.	Female	Adult	The ceiling of a PHC facility	2021	AlphaCoV	NT	NT	OM869903
NG014_NGR	*Chaerephon* sp.	Female	Adult	The ceiling of a PHC facility	2021	AlphaCoV	NT	NT	OM869904
NG017_NGR	*Chaerephon* sp.	Male	Adult	The ceiling of a PHC facility	2021	AlphaCoV	NT	NT	OM869905
NG019_NGR	*Chaerephon* sp.	Female	Adult	The ceiling of a PHC facility	2021	AlphaCoV	NT	NT	OM869906
NG020_NGR	*Chaerephon* sp.	Female	Adult	The ceiling of a PHC facility	2021	AlphaCoV	NT	NT	OM869907
NG022_NGR	*Chaerephon* sp.	Female	Adult	The ceiling of a PHC facility	2021	AlphaCoV	NT	NT	OM869908
NG024_NGR	*Chaerephon* sp.	Female	Adult	The ceiling of a PHC facility	2021	AlphaCoV	NT	NT	OM869909
NG033_NGR	*Chaerephon* sp.	Female	Adult	The ceiling of a PHC facility	2021	AlphaCoV	NT	NT	OM869910
NG044_NGR	*Chaerephon* sp.	Female	Juvenile	The ceiling of a PHC facility	2021	AlphaCoV	NT	NT	OM869911
NG046_NGR	*Chaerephon* sp.	Female	Juvenile	The ceiling of a PHC facility	2021	AlphaCoV	NT	NT	OM869912

Note: 1 = coinfection with AlphaCoV and BetaCoV; -ve = negative; NT = not tested due to low sample volume; PHC = primary health care facility; OAU = Obafemi Awolowo University; CER = Centre for Energy Research.

## Data Availability

Genome sequences of bat coronaviruses reported in this study have been deposited in GenBank, as shown in Table 2. Genome sequences from bat species identified in this study have been deposited in GenBank under accession numbers ON716456-ON716474, ON721268-ON721269, and ON748933-0N748941.
